# ﻿Two new species of *Colletotrichum* (Glomerellaceae, Glomerellales) causing walnut anthracnose in Beijing

**DOI:** 10.3897/mycokeys.99.106812

**Published:** 2023-09-07

**Authors:** Lin Zhang, Yue-Qi Yin, Li-Li Zhao, Yu-Qing Xie, Jing Han, Ying Zhang

**Affiliations:** 1 School of Ecology and Nature Conservation, Beijing Forestry University, Beijing 100083, China Beijing Forestry University Beijing China; 2 School of Biological Science and Technology, Beijing Forestry University, Beijing 100083, China Beijing Forestry University Beijing China; 3 School of Forestry, Beijing Forestry University, Beijing 100083, China Beijing Forestry University Beijing China

**Keywords:** Anthracnose, multi-gene phylogeny, pathogenicity, walnut

## Abstract

*Colletotrichum* species are plant pathogens, saprobes and endophytes on various plant hosts. It is regarded as one of the 10 most important genera of plant pathogens in the world. Walnut anthracnose is one of the most severe diseases affecting walnut productivity and quality in China. In this study, 162 isolates were obtained from 30 fruits and 65 leaf samples of walnut collected in Beijing, China. Based on morphological characteristics and DNA sequence analyses of the concatenated loci, namely internal transcribed spacer (ITS), glyceraldehyde-3-phosphate dehydrogenase (*GAPDH*), actin (*ACT*), chitin synthase 1 (*CHS-1*) and beta-tubulin (TUB2), these isolates were identified as two novel species of *Colletotrichum*, i.e. *C.juglandicola* and *C.peakense*. Koch’s postulates indicated that both *C.juglandicola* and *C.peakense* could cause anthracnose in walnut.

## ﻿Introduction

Walnut (*Juglansregia* L.), a deciduous tree, is an essential woody nut and oil crop cultivated worldwide (Da Lio et al. 2018). Walnut fruit is rich in linolenic acid and lacking cholesterol and ranks first amongst the world’s “four major dried fruits” (Pei and Lu 2011). Walnut cultivation in China has a history of more than two thousand years and is China’s “woody grain and oil” strategic tree species (Guo 2016; Liu et al. 2021). The walnut productivity in China contributed 47% of the global production in 2017 and ranked first worldwide since 2017 (Liu et al. 2021).

*Colletotrichum* Corda (Glomerellaceae, Glomerellales, Sordariomycetes) was introduced, based on the morphological feature of the conidiomata with setae and *Colletotrichumlineola* Corda was assigned as the generic type (Corda 1831). The sexual morph of *Colletotrichum* was previously known as the genera *Gnomoniopsis* and *Glomerella* (Stoneman 1898; von Schrenk and Spaulding 1903; Marin-Felix et al. 2017). With the implementation of “one fungus one name” nomenclature, *Colletotrichum* has been chosen to represent this genus, based on priority (Réblová et al. 2016). *Colletotrichum* was characterised by acervular conidiomata, often with setae, producing cylindrical or crescent-shaped conidia and by the formation of appressoria (Sutton 1992; Marin-Felix et al. 2017). More than 1,000 epithets have been accommodated within *Colletotrichum* (http://www.indexfungorum.org, accessed 20 March 2023), while about 300 species have DNA sequence data to support their taxonomic status within *Colletotrichum*. Sixteen species complexes have been recognised within *Colletotrichum*, with *C.gloeosporioides* species complex as the largest one, which occupies more than 18% of all the recognised taxa of *Colletotrichum* (Marin-Felix et al. 2017; Damm et al. 2019; Jayawardena et al. 2020; Bhunjun et al. 2021; Mu et al. 2021; Talhinhas and Baroncelli 2021; Alizadeh et al. 2022; Liu et al. 2022; Tsushima and Shirasu 2022; Zheng et al. 2022).

*Colletotrichum* spp. comprised important plant pathogens, while others are endophytes or saprobes (Cannon et al. 2012; Hyde et al. 2014; Jayawardena et al. 2016). Some *Colletotrichum* species have been reported causing anthracnose disease on various host plants (Hyde et al. 2014; Nilsson et al. 2014). For instance, the causal agents of ginseng anthracnose were *C.lineola* and *C.panacicola* in China (Liu et al. 2020). Anthracnose of *Pyrus* spp. was caused by 12 *Colletotrichum* species in China, viz. *C.aenigma*, *C.citricola*, *C.conoides*, *C.fioriniae*, *C.fructicola*, *C.gloeosporioides*, *C.karstii*, *C.plurivorum*, *C.siamense*, *C.wuxiense*, *C.jinshuiense* and *C.pyrifoliae* (Fu et al. 2019). Quite a few species of *Collectotrichum* have been reported to be causing walnut anthracnose disease, which has resulted in a considerable reduction in walnut production worldwide (Zhu et al. 2014; Wang et al. 2016; Da Lio et al. 2018). For instance, the causal agent of walnut anthracnose identified as *Colletotrichum* spp. led to 50–70% losses, with some walnut orchards experiencing 100% losses in nut production in France (Giraud and Verhaeghe 2015). *Colletotrichumnymphaeae* caused anthracnose in walnut in Brazil, destroyed approximately 50% of the fruits and the incidence was higher in rainy and hot summers (Savian et al. 2019).

In China, 12 *Colletotrichum* species have been reported causing walnut anthracnose. Sever walnut anthracnose occurred in the orchards of Shandong Province, with the causal agents *C.gloeosporioides* sensu lato, *C.siamense*, *C.fructicola* and *C.viniferum* (Zhu et al. 2014; Wang et al. 2017, 2018; He et al. 2019). The walnut leaf anthracnose caused by *C.fioriniae* led to severe loss in nut production in Hechi, Guangxi Province (Zhu et al. 2015). In addition, *Colletotrichumaenigma* caused severe fruit anthracnose in Hebei Province (Wang et al. 2021). *Colletotrichumnymphaeae* caused walnut branches anthracnose in Gansu Province (Ma et al. 2022). *Colletotrichumgloeosporioides*, *C.kahawae*, *C.nymphaeae*, *C.godetiae*, *C.fioriniae* and *C.juglandis* caused leaf spots of walnut in Hubei Province (Wei et al. 2022). *Colletotrichumgodetiae* caused severe anthracnose of walnut in Shaanxi Province and Yunnan Province with diseased fruits over 60% in the orchard (Wang et al. 2023). Li et al. (2023) collected 900 walnut leaves and 300 fruits samples from seven districts of Beijing and 377 isolates of *Colletotrichum* spp. were identified into six species, namely *C.aenigma*, *C.fructicola*, *C.gloeosporioides*, *C.siamense*, *C.liaoningense* and *C.sojae*. All of these six species caused anthracnose of walnut and *C.gloeosporioides* showed the highest virulence.

In the course of an ongoing survey of pathogenic fungi of walnuts in China initiated in 2021, the symptoms on the fruits included round brown spots in the early stage that later turned black. As environmental humidity increased, the spots were covered with orange-red conidiomata. Some spots were merged into large necrotic areas, causing the whole fruit to blacken and rot, resulting in fruit drop. The symptoms on the leaves included nearly round or irregular black or brown spots and gradually withering. A total of 162 isolates were obtained from 30 fruits and 65 leaf samples of walnut collected in the suburb area of Beijing. Their taxonomic status was evaluated, based on morphological characteristics and DNA sequence comparisons and pathogenicity were evaluated by proving Koch’s postulates.

## ﻿Materials and methods

### ﻿Sample collection and fungal isolation

Thirty fruits and sixty-five leaf samples exhibiting anthracnose were collected from the suburb area of Beijing, China, in August, 2021. Specimens were transferred to the laboratory and kept in a freezer. Fragments (0.5 × 0.5 cm) were cut aseptically from the margin of the disease lesion and surface-sterilised with 75% ethanol for 30 s, rinsed three times with sterile distilled water, dried on sterilised filter paper and incubated on malt extract agar (MEA; 2%) for isolation of fungal strains (Zhao et al. 2019a). Petri dishes were incubated in the dark at 25 °C until the fungal colonies were observed. Hyphal tips resembling *Colletotrichum* colonies were transferred to Petri dishes with MEA. Isolates grown on MEA in the dark were kept at 25 °C to determine colony characteristics.

### ﻿Morphological characterisation

To assess the colony characteristics, mycelial plugs (8 mm in diameter) were transferred from the growing edges of 7-day-old colonies on to PDA and MEA and incubated at 25 °C under dark conditions (Liu et al. 2017a; Zhao et al. 2019a). Colony diameters were measured after 7 days’ incubation (Liu et al. 2017b) and were used to calculate hyphal growth rate (Mo et al. 2018). Morphology and colony characteristics were determined following Damm et al. (2009, 2014) and Choi et al. (2011). Appressoria were induced on slide cultures, according to Weir et al. (2012). The shape, colour and size of conidia, conidiophores, setae, conidiogenous cells and appressoria were measured by at least 20 measurements using a microscope (Nikon Eclipse E600) (Zhao et al. 2019b). Fungal isolates and specimens were deposited at Beijing Forestry University, with duplicates at the China General Microbiological Culture Collection Center (**CGMCC**) and the Mycological Herbarium of the Institute of Microbiology, Chinese Academy of Sciences (**HMAS**).

### ﻿DNA extraction, PCR amplification and sequencing

DNA was extracted from mycelia grown on MEA plates with CTAB plant genome DNA fast extraction kit (Aidlab Biotechnologies Co., Ltd, Beijing, China) and stored at -20 °C until further use. Five loci, including the 5.8S nuclear ribosomal gene with the two flanking internal transcribed spacers (ITS), a 200-bp intron of the glyceraldehyde-3-phosphate dehydrogenase (*GAPDH*), partial actin (*ACT*), beta-tubulin (*TUB2*) and chitin synthase 1 (*CHS-1*), were amplified using the primer pairs ITS1/ITS4 (White et al. 1990; Gardes and Bruns 1993), GDF1/GDR1 (Guerber et al. 2003), ACT-512F/ACT-783R (Carbone and Kohn 1999), T1/Bt2b (Glass and Donaldson 1995; O’Donnell and Cigelnik 1997) and CHS-79F/CHS-345R (Carbone and Kohn 1999), respectively.

PCR amplification and sequencing followed the protocols of Liu et al. (2017a). PCR amplicons were purified and sequenced at BGI Tech Solutions (Beijing Liuhe) Co., Limited (Beijing, China). Forward and reverse were assembled to obtain a consensus sequence with DNAMAN (v. 6.0.3.99; Lynnon Biosoft). Sequences generated in this study were deposited in GenBank (Table 1).

**Table 1.** GenBank accession numbers of isolates included in this study (newly-generated sequences are in bold). * = ex-type or authentic culture, (*) = ex-type or authentic culture of synonymised taxon and N/A = not available. Newly-generated sequences are indicated in bold.

Taxon	Isolate designation	Host	Location	GenBank accession number(s)				
				ITS	GAPDH	CHS-1	ACT	TUB2
*Colletotrichumaenigma*	ICMP 18608*	*Perseaamericana*	Israel	JX010244	JX010044	JX009774	JX009443	JX010389
*C.aeschynomenes*	ICMP 17673*	*Aeschynomenevirginica*	USA	JX010176	JX009930	JX009799	JX009483	JX010392
*C.alatae*	CBS 304.67*	*Dioscoreaalata*	India	JX010190	JX010190	JX009837	JX009471	JX010383
*C.alienum*	ICMP 12071*	*Malusdomestica*	New Zealand	JX010251	JX010028	JX009882	JX009572	JX010411
*C.aotearoa*	ICMP 18537*	*Coprosma* sp.	New Zealand	JX010205	JX010005	JX009853	JX009564	JX010420
*C.arecicola*	CGMCC 3.19667*	*Arecacatechu*	China	MK914635	MK935455	MK935541	MK935374	MK935498
*C.artocarpicola*	MFLUCC 18-1167*	*Artocarpusheterophyllus*	Thailand	MN415991	MN435568	MN435569	MN435570	MN435567
*C.asianum*	ICMP 18580*	*Coffeaarabica*	Thailand	FJ972612	JX010053	JX009867	JX009584	JX010406
*C.australianum*	VPRI 43075*	*Citrussinensis*	Australia	MG572138	MG572127	MW091987	MN442109	MG572149
*C.boninense*	MAFF 305972* = CBS 123755	Crinumasiaticumvar.sinicum	Japan	JQ005153	JQ005240	JQ005327	JQ005501	JQ005588
*C.camelliae*	CGMCC 3.14925	*Camelliasinensis*	China	KJ955081	KJ954782	MZ799255	KJ954363	KJ955230
*C.changpingense*	MFLUCC 15-0022 = CGMCC 3.17582*	*Rhizome of Fragaria × ananass*	China	KP683152	MZ664048	KP852449	KP683093	MZ673952
*C.chiangmaiense*	MFLUCC 18-0945*	*Magnoliagarrettii*	Thailand	MW346499	MW548592	MW623653	MW655578	N/A
*C.chrysophilum*	CMM 4268*	*Musa* sp.	Brazil	KX094252	KX094183	KX094083	KX093982	KX094285
*C.cigarro*	ICMP 18539*	*Oleaeuropaea*	Australia	JX010230	JX009966	JX009800	JX009523	JX010434
*C.citrulli*	CAASZT54	*Citrulluslanatus*	China	MZ475134	OL456686	OL901154	OL449284	OL456645
	CAASZT52	*Citrulluslanatus*	China	MZ475133	OL456685	OL901153	OL449283	OL456644
*C.clidemiae*	ICMP 18658*	*Clidemiahirta*	USA, Hawaii	JX010265	JX009989	JX009877	JX009537	JX010438
*C.cobbittiense*	BRIP 66219*	*Cordylinestricta × C.australis*	Australia	MH087016	MH094133	MH094135	MH094134	MH094137
*C.conoides*	CGMCC 3.17615	*Chilipepper*	China	KP890168	KP890162	KP890156	KP890144	KP890174
*C.cordylinicola*	ICMP 18579*	*Cordylinefruticosa*	Thailand	JX010226	JX009975	JX009864	HM470235	JX010440
*C.dimorphum*	CGMCC 3.16083*	*Ageratinaadenophora*	China	OK030867	OK513670	OK513566	OK513606	OK513636
	YMF 1.07303	*Ageratinaadenophora*	China	OK030866	OK513669	OK513565	OK513605	OK513635
*C.dracaenigenum*	MFLUCC 19-0430*	*Dracaena* sp.	Thailand	MN921250	MT215577	MT215575	MT313686	N/A
*C.endophyticum*	MFLUCC 13-0418*	*Pennisetumpurpureum*	Thailand	KC633854	KC832854	MZ799261	KF306258	MZ673954
*C.fici-septicae*	MFLU 19-27708*	*Capsicumannuum*	China	KP145441	KP145413	KP145385	KP145329	KP145469
*C.fructicola*	ICMP 18581*	*Coffeaarabica*	Thailand	JX010165	JX010033	JX009866	FJ907426	JX010405
*C.fructivorum*	CBS 133125*	*Vacciniummacrocarpon*	Burlington	JX145145	MZ664047	MZ799259	MZ664126	JX145196
*C.gloeosporioides*	IMI 356878* = ICMP 17821	*Citrussinensis*	Italy	JX010152	JX010056	JX009818	JX009531	JX010445
	CBS 273.51(*) = ICMP 19121	*Citruslimon*	Italy	JX010148	JX010054	JX009903	JX009558	N/A
	DAR 76936 = ICMP 18738	*Caryaillinoinensis*	Australia	JX010151	JX009976	JX009797	JX009542	N/A
	ICMP12939	*Citrus* sp.	New Zealand	JX010149	JX009931	JX009747	JX009462	N/A
	CBS 119204 = ICMP 18678	*Puerarialobata*	USA	JX010150	JX010013	JX009790	JX009502	N/A
	ICMP 12066	*Ficus* sp.	New Zealand	JX010158	JX009955	JX009888	JX009550	N/A
	ICMP 18730	*Citrus* sp.	New Zealand	JX010157	JX009981	JX009861	JX009548	N/A
*C.gloeosporioides*	ICMP 12938	*Citrussinensis*	New Zealand	JX010147	JX009935	JX009746	JX009560	N/A
	ICMP 18694	*Mangiferaindica*	South Africa	JX010155	JX009980	JX009796	JX009481	N/A
	ICMP 18695	*Citrus* sp.	USA	JX010153	JX009979	JX009779	JX009494	N/A
	ICMP 18697	*Vitisvinifera*	USA	JX010154	JX009987	JX009780	JX009557	N/A
*C.grevilleae*	CBS 132879*	*Grevillea* sp.	Italy	KC297078	KC297010	KC296987	KC296941	KC297102
*C.grossum*	CGMCC 3.17614*	*Chilipepper*	China	KP890165	KP890159	KP890153	KP890141	KP890171
*C.hebeiense*	MFLUCC 13-0726*	*Vitisvinifera*	China	KF156863	KF377495	KF289008	KF377532	KF288975
*C.hederiicola*	MFLU 15-0689*	*Hederahelix*	Italy	MN631384	N/A	MN635794	MN635795	N/A
*C.helleniense*	CBS 142418*	*Poncirustrifoliata*	Greece, Arta	KY856446	KY856270	KY856186	KY856019	KY856528
*C.henanense*	CGMCC 3.17354*	*Camelliasinensis*	China	KJ955109	KJ954810	MZ799256	KM023257	KJ955257
*C.horii*	NBRC 7478*	*Diospyroskaki*	Japan	GQ329690	GQ329681	JX009752	JX009438	JX010450
*C.hystricis*	CBS 142411*	*Citrushystrix*	Italy, Catania	KY856450	KY856274	KY856190	KY856023	KY856532
*C.jiangxiense*	CGMCC 3.17361*	*Camelliasinensis*	China	KJ955149	KJ954850	MZ799257	KJ954427	OK236389
*C.kahawae*	IMI 319418*	*Coffeaarabica*	Kenya	JX010231	JX010012	JX009813	JX009452	JX010444
*C.ledongense*	CGMCC3.18888*	*Quercuspalustris*	China	MG242008	MG242016	MG242018	MG242014	MG242010
*C.makassarense*	CBS 143664*	*Capsicumannuum*	Indonesia	MH728812	MH728820	MH805850	MH781480	MH846563
*C.mengyinense*	SAUCC0702*	*Rosachinensis*	China	MW786742	MW846240	MW883686	MW883695	MW888970
*C.musae*	CBS 116870*	*Musa* sp.	USA	JX010146	JX010050	JX009896	JX009433	HQ596280
*C.nanhuaensis*	CGMCC 3.18962*	*Ageratinaadenophora*	China	OK030870	OK513673	OK513569	OK513609	OK513639
	YMF 1.04990	*Ageratinaadenophora*	China	OK030871	OK513674	OK513570	OK513610	OK513640
*C.nupharicola*	CBS 470.96*	Nupharluteasubsp.polysepala	USA	JX010187	JX009972	JX009835	JX009437	JX010398
*C.pandanicola*	MFLUCC 17-0571*	*Pandanaceae*	Thailand	MG646967	MG646934	MG646931	MG646938	MG646926
*C.perseae*	CBS 141365*	*Avocado*	Israel	KX620308	KX620242	MZ799260	KX620145	KX620341
*C.proteae*	CBS 132882*	*Protea* sp.	South Africa	KC297079	KC297009	KC296986	KC296940	KC297101
*C.pseudotheobromicola*	MFLUCC 18-1602*	*Prunusavium*	China	MH817395	MH853675	MH853678	MH853681	MH853684
*C.psidii*	CBS 145.29*	*Psidium* sp.	Italy	JX010219	JX009967	JX009901	JX009515	JX010443
*C.queenslandicum*	ICMP 1778*	*Caricapapaya*	Australia	JX010276	JX009934	JX009899	JX009447	JX010414
*C.rhexiae*	CBS 133134*	*Rhexiavirginica*	Sussex	JX145128	MZ664046	MZ799258	MZ664127	JX145179
*C.salsolae*	ICMP 19051*	*Salsolatragus*	Hungary	JX010242	JX009916	JX009863	JX009562	JX010403
*C.siamense*	ICMP 18578*	*Coffeaarabica*	Thailand	JX010171	JX009924	JX009865	FJ907423	JX010404
*C.syzygicola*	MFLUCC 10-0624*	*Syzygiumsamarangense*	Thailand	KF242094	KF242156	N/A	KF157801	KF254880
*C.tainanense*	CBS 143666*	*Capsicumannuum*	Taiwan	MH728818	MH728823	MH805845	MH781475	MH846558
*C.temperatum*	CBS 133122*	*Vacciniummacrocarpon*	Bronx	JX145159	MZ664045	MZ799254	MZ664125	JX145211
*C.tengchongense*	YMF 1.04950	*Isoetessinensis*	China	OL842169	OL981264	OL981290	OL981238	N/A
*C.theobromicola*	CBS 124945*	*Theobromacacao*	Panama	JX010294	JX010006	JX009869	JX009444	JX010447
*C.ti*	ICMP 4832*	*Cordyline* sp.	New Zealand	JX010269	JX009952	JX009898	JX009520	JX010442
*C.tropicale*	CBS 124949*	*Theobromacacao*	Panama	JX010264	JX010007	JX009870	JX009489	JX010407
*C.viniferum*	GZAAS 5.08601*	*Vitisvinifera* cv. *Shuijing*	China	JN412804	JN412798	N/A	JN412795	N/A
*C.vulgaris*	YMF 1.04940	*Hippurisvulgaris*	China	OL842170	OL981265	OL981291	OL981239	N/A
*C.wuxiense*	CGMCC 3.17894*	*Camelliasinensis*	China	KU251591	KU252045	KU251939	KU251672	KU252200
*C.xanthorrhoeae*	BRIP 45094*	*Xanthorrhoeapreissii*	Australia	JX010261	JX009927	JX009823	JX009478	JX010448
*C.xishuangbannaense*	MFLUCC 19-0107*	*Magnolialiliifera*	China	MW346469	MW537586	MW660832	MW652294	N/A
*C.yulongense*	CFCC 50818*	Vacciniumdunalianumvar.urophyllum	China	MH751507	MK108986	MH793605	MH777394	MK108987
*C.yunanjiangensis*	CGMCC 3.18964*	*Ageratinaadenophora*	China	OK030885	OK513686	OK513583	OK513620	OK513649
***C.peakense***	CGMCC3.24308*	***Juglansregia***	**China**	**OQ263017**	**OQ282975**	**OR004795**	**OQ282968**	**OQ282982**
	**CGMCC3.24307**	***Juglansregia***	**China**	**OQ263016**	**OQ282974**	**OR004794**	**OQ282967**	**OQ282981**
***C.juglandicola***	CGMCC3.24312*	***Juglansregia***	**China**	**OQ263015**	**OQ282973**	**OR004793**	**OQ282966**	**OQ282980**
	**CGMCC3.24313**	***Juglansregia***	**China**	**OQ263018**	**OQ282977**	**OR004797**	**OQ282970**	**OQ282984**
	**CGMCC3.24310**	***Juglansregia***	**China**	**OQ263020**	**OQ282979**	**OR004799**	**OQ282972**	**OQ282986**
	**CGMCC3.24309**	***Juglansregia***	**China**	**OQ263021**	**OQ282978**	**OR004798**	**OQ282971**	**OQ282985**
	**CGMCC3.24311**	***Juglansregia***	**China**	**OQ263019**	**OQ282976**	**OR004796**	**OQ282969**	**OQ282983**

### ﻿Phylogenetic analysis

DNA sequences of concatenated *ACT*, *CHS-1*, *GAPDH*, ITS and *TUB2* loci were analysed to investigate the phylogenetic relationships amongst *Colletotrichum* species with DNA sequences available from GenBank (http://www.ncbi.nlm.nih.gov/genbank/), as well as the sequences generated herein (Table 1). Multiple sequences were aligned using the MAFFT v.7.110 (http://mafft.cbrc.jp/alignment/server/) and adjusted manually in MEGA v.7.0 (Kumar et al. 2016). Gaps were manually adjusted to optimise the alignment (Tamura et al. 2013).

Phylogenetic analyses of Maximum Likelihood (ML), Bayesian Inference (BI) and Maximum Parsimony (MP) were performed. ML analyses were constructed on the RAxML-HPC BlackBox 8.2.10 (Stamatakis 2014) using the GTR+GAMMA model with 1000 bootstrap replicates. The Bayesian phylogenetic analysis was performed using a Markov Chain Monte Carlo (MCMC) algorithm in MrBayes v. 3.2.6 (Ronquist et al. 2012). Four MCMC chains were run from random trees for 2,000,000 generations and trees were sampled by each 1,000^th^ generation. The first 25% of the trees of MCMC sampling were discarded as burn-in and posterior probabilities (PP) were determined from the remaining trees. Maximum Parsimony (MP) analysis, based on the concatenated dataset, was conducted in PAUP* v. 4.0b10 with the default options (Swofford 2002). Ambiguous regions in the alignment were excluded and gaps were treated as missing data. Clade stability was evaluated in a bootstrap analysis with 1,000 replicates with Maxtrees set to 1,000 and other default parameters implemented in PAUP* (Hillis and Bull 1993). Other measurements calculated parsimony scores including consistency index (CI), rescaled consistency (RC), homoplasy index (HI) and retention index (RI). The phylogenetic trees were configured in FigTree v. 1.4.4 (http://tree.bio.ed.ac.uk/software/figtree) and edited using Adobe Illustrator CC2020 (Adobe Systems Inc., USA).

Sequences were analysed using the GCPSR model by performing a pairwise homoplasy index (PHI) test as described by Quaedvlieg et al. (2014) for the phylogenetically close, but not clearly delimited species. The PHI test was performed in SplitsTree v. 4.19.1 (Huson and Bryant 2006) to determine the recombination level within phylogenetically closely-related species using a five-locus concatenated dataset (*ACT*, *CHS-1*, *GAPDH*, ITS and *TUB2*). If the resulting pairwise homoplasy index was below a 0.05 threshold (Фw < 0.05), it was indicative of significant recombination in the dataset. The relationship between closely-related species was visualised by constructing a split graph (Chethana et al. 2021; Jayawardena et al. 2021; Peng et al. 2023).

### ﻿Pathogenicity tests and virulence on walnut tissues

All isolated species were tested for their pathogenicity on walnut fruits and leaves. Isolates of all species were incubated on MEA plates for 7 days prior to inoculation. Spore suspension of isolates of *Colletotrichumjuglandicola* (CGMCC3.24312) and *Colletotrichumpeakense* (CGMCC3.24308) obtained in this study were used for pathogenicity testing.

The pathogenicity test was performed on detached living walnut fruits and leaves. Briefly, fruits and leaves were washed with sterilised water and surface sterilised with 75% ethanol for 1 min. The fruits and leaves were inoculated using the spore suspension and non-wound inoculation methods (Fu et al. 2019; Zhao et al. 2019b). For the spore suspension and non-wound method, an aliquot of 20 μl of spore suspension (1.0 × 10^6^ conidia per ml) was inoculated on to fruits and leaves without wounding them. Eight replicates were used for each isolate. Sterilised water was used as the negative control. The inoculated detached fruits and leaves were incubated under 25 °C with 12/12 h light/dark photoperiod. Pathogenicity was determined by measuring the lesion length of fruits and leaves after 10 days’ incubation. Mean comparisons were conducted using Tukey’s honest significant difference (HSD) test (α = 0.05) in R (Version 3.2.2, R Inc. Auckland, NZL).To fulfil Koch’s postulates, small pieces of infected tissue were plated on to MEA to re-isolate the fungal isolates, which were identified, based on morphology and DNA sequences.

## ﻿Results

### ﻿Phylogenetic analyses

**Figure 1.**
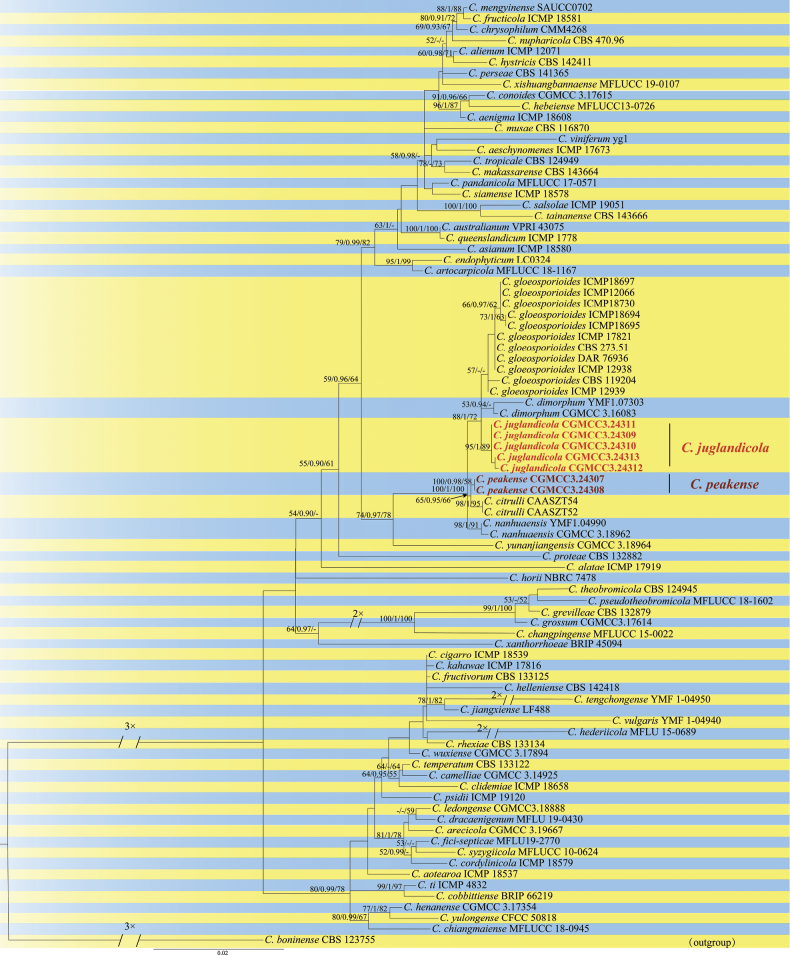
Phylogenetic tree of Maximum Likelihood analyses of 86 isolates in the *C.gloeosporioides* species complex. The species *C.boninense* (CBS 123755) was selected as an outgroup. The tree was built using concatenated sequences of *ACT*, *CHS-1*, *GAPDH*, ITS and *TUB2* genes. RAxML bootstrap support values (ML ≥ 50%), Bayesian posterior probability (PP ≥ 0.90) and MP bootstrap support values (ML ≥ 50%) are shown at the nodes (ML/PP/MP).

The concatenated *ACT*, *CHS-1*, *GAPDH*, ITS and *TUB2* dataset (1,948 characters with 369 parsimony-informative characters) from 79 in-group isolates of *Colletotrichumgloeosporioides* species complex was used for phylogenetic analysis. The outgroup taxon was *C.boninense* CBS 123755. The heuristic search with random addition of taxa (1,000 replicates) generated 5,000 most parsimonious trees (Length = 1,313, CI = 0.673, HI = 0.327, RI = 0.854, RC = 0.575). The topologies obtained from the Maximum Parsimony, Maximum Likelihood and Bayesian analysis were comparable. In three analyses (ML, BI and MP), *Colletotrichumjuglandicola* and *Colletotrichumpeakense* are consistently sibling to all other species of *C.gloeosporioides* species complex (95/1/89 and 100/0.98/58) (Fig. 1). Additionally, only the ML tree is presented here, with ML, BI and MP values plotted on the branches (Fig. 1).

**Figure 2.**
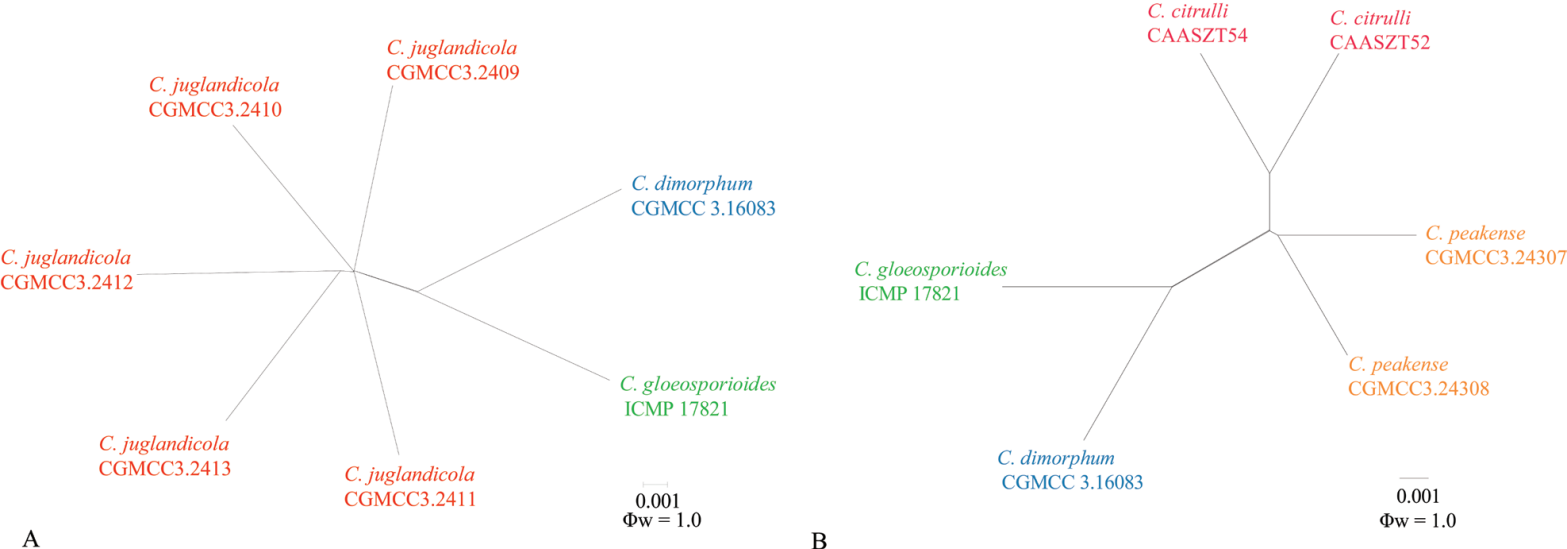
The result of the pairwise homoplasy index (PHI) tests of closely-related species using both LogDet transformation and splits decomposition **A, B** the PHI of *C.juglandicola* (**A**) or *C.peakense* (**B**) and their phylogenetically related isolates or species, respectively. PHI test value (Φw) < 0.05 indicate significant recombination within the dataset.

To exclude the possibility that species delimitation might be interfered by recombination amongst the genes used for phylogenetic analyses, the multi-locus (*ACT*, *CHS-1*, *GAPDH*, ITS and *TUB2*) concatenated datasets were subjected to two PHI tests (Fig. 2) to determine the recombination level within phylogenetically closely-related species. The results showed that no significant recombination events were observed between *Colletotrichumjuglandicola* and phylogenetically related isolates or species (*C.gloeosporioides* and *C.dimorphum*) (Fig. 2A) and between *C.peakense* and phylogenetically related species (*C.citrulli*, *C.gloeosporioides* and *C.dimorphum*) (Fig. 2B).

### ﻿Taxonomy

#### ﻿ Colletotrichum juglandicola

Taxon classificationFungiGlomerellalesGlomerellaceae

Y. Zhang ter. & L. Zhang sp. nov.

2B34B376-58C5-5681-B780-3BAC350A1FD6

MycoBank No: 848731

Fig. 3

##### Etymology.

Named from “*Juglans*”, in reference to the host genus.

##### Description.

***Sexual morph*** not observed. ***Asexual morph*** developed on MEA. ***Conidiomata*** acervular, yellow to light brown, bearing conidial masses. ***Conidiophores*** hyaline, smooth-walled, septate, branched. ***Setae*** medium to dark brown, smooth to finely verruculose close to the tip, the tip rounded, 1–3 aseptate, 60–107.2 μm long. ***Conidiogenous cells*** 19.5–38.9 × 2.8–3.9 μm (mean SD = 28.6 ± 1.2 × 3.3 ± 0.1 μm, n = 20), subcylindrical, straight to curved. ***Conidia*** 14.6–20.0 × 4.2–6.6 μm (mean SD = 17.1 ± 1.0 × 5.2 ± 0.4 μm, L/W radio = 3.3, n = 100), hyaline, smooth-walled, subcylindrical, both ends round, 1–3-guttulate, contents granular. ***Appressoria*** 5–8.3 × 3.3–6.7 μm (mean SD = 6.3 ± 0.2 × 5.2 ± 0.2 μm, L/W radio = 1.2, n = 20), medium to dark brown, variable in shape, often smooth-walled, subglobose, ovate to broadly elliptical in outline.

##### Culture characteristics.

Colonies on MEA, flat, with entire margin, hyaline, 65–72 mm diam. in 7 d. The colonies are round, white, the edges are flat and the aerial hyphae are lush. Myxospores are orange. The colony diameter reached 63–65 mm on PDA. The colonies are round, green-grey, the edges are flat and the aerial hyphae are lush.

##### Additional specimens examined.

China, Beijing, Changping District, Heishanzhai Village, from leaf of *Juglansregia* L., Y. Zhang and L. Zhang, 26 August 2021 (holotype HSG826-P5; ex-type living culture: CGMCC3.24312). CHINA, Beijing, Huairou District, Shuichangcheng Village, from leaf of *Juglansregia* L., Aug 2021, Y. Zhang and L. Zhang (Paratype SCCY826-22; living culture: CGMCC3.24313). CHINA, Beijing, Haidian District, Jiufeng Village, from fruit of *Juglansregia* L., Aug 2021, Y. Zhang and L. Zhang (Paratype JFG826-P4; living culture CGMCC3.24311). China, Beijing, Changping District, Yanshou Village, from fruit of *Juglansregia* L., Aug 2021, Y. Zhang and L. Zhang (Paratype YSG826-R1; living culture CGMCC3.24309). CHINA, Beijing, Changping District, Yanshou Village, from leaf of *Juglansregia* L., Aug 2021, Y. Zhang and L. Zhang (Paratype YSY826-2: living culture CGMCC3.24310).

**Figure 3.**
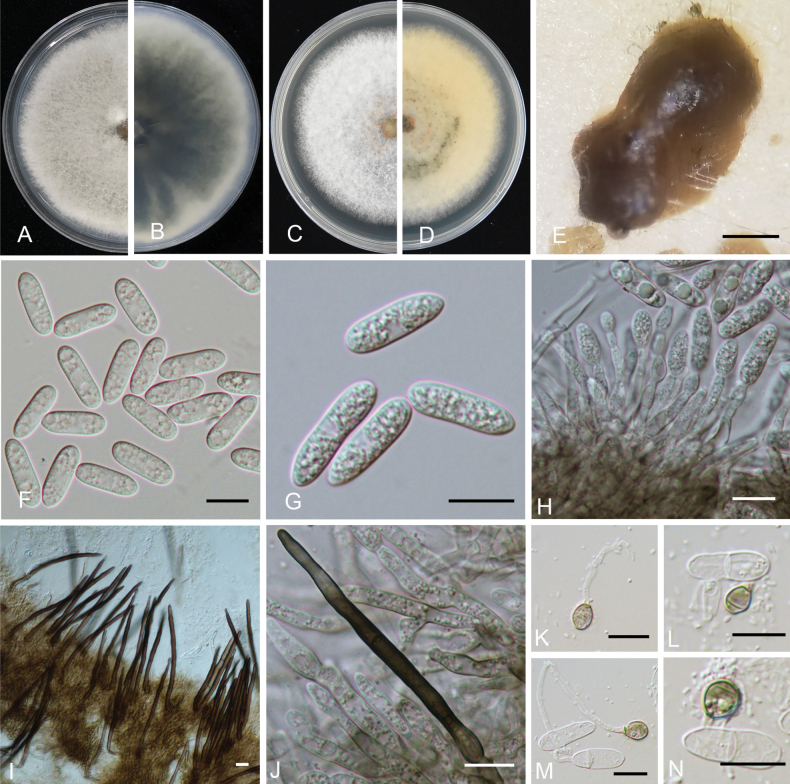
*Colletotrichumjuglandicola* (from ex-type CGMCC3.24312) **A, B** colonies and reverse after 7 days on PDA medium **C, D** colonies and reverse after 7 days on MEA medium **E** conidiomata **F, G** conidia **H** conidiophores **I, J** setae **K–N** appressoria. Scale bars: 500 μm (**E**); 10 μm (**F–N**).

##### Notes.

Phylogenetic analysis of a concatenated five loci dataset indicated that the clade of *Colletotrichumjuglandicola* nested in the clade of *C.gloeosporioides* species complex and was closely related, but independent to *C.citrulli*, *C.dimorphum*, *C.gloeosporioides* and *C.nanhuaensis* (Cannon et al. 2008; Guo et al. 2022; Yu et al. 2022). *Colletotrichumcitrulli* was reported from *Citrulluslanatus* (Cucurbitaceae) in China (Guo et al. 2022). Morphologically, *C.juglandicola* differed from *C.citrulli* by having longer conidia and setae and smaller appressoria (Table 2) (Guo et al. 2022). *Colletotrichumdimorphum* was reported from *Ageratinaadenophora* (Asteraceae) in China (Guo et al. 2022). Morphologically, *C.juglandicola* differed from *C.dimorphum* by having setae, shorter appressoria and longer conidia (Yu et al. 2022) (Table 2). Morphologically, *C.juglandicola* differed from *C.gloeosporioides* or *C.nanhuaensis* by having longer conidia (Cannon et al. 2008; Guo et al. 2022) (Table 2). *Colletotrichumnanhuaensis* was reported from *Ageratinaadenophora* (Asteraceae) in China (Guo et al. 2022) (Table 2). The PHI test (Φw = 1.0) detected no significant recombination between related isolates or species (Fig. 2A).

**Table 2.** Morphological comparison of species in the gloeosporioides species complex.

Species	Type	Hosts	Distribution	Conidia (Mean ± SD) (μm)	Appressoria (μm)	Setae (μm)	Reference
*Colletotrichumcitrulli*	Holotype	*Citrulluslanatus*	China	16.2 ± 0.9 × 5.6 ± 0.5	8.0–12.0 × 6.0–10.0	42.0–79.0	Guo et al. (2022)
*C.aenigma*	Holotype	*Perseaamericana*	Israel	14.5 × 6.1	6.0–10.0	Not observed	Weir et al. (2012)
*C.dimorphum*	Holotype	*Ageratinaadenophora*	China	14.6 ± 2 × 4.8 ± 0.7	5.7–10.6 × 5.0–9.0	Not observed	Yu et al. (2022)
*C.fructicola*	Holotype	*Coffeaarabica*	Thailand	11.5 ± 1.0 × 3.6 ± 0.3	4.3–9.7 × 3.7–7.3	Not observed	Prihastuti et al. (2009)
*C.gloeosporioides*	epitype	*Citrussinensis*	Italy	14.4 × 5.6	7.2–8.6 × 4.7–6.0	40.0–120.0	Cannon et al. (2008)
***C.juglandicola***	**Holotype**	***Juglansregia* L.**	**China**	**17.1 ± 1.0 × 5.2 ± 0.4**	**5–8.3 × 3.3–6.7**	60.0–107.2	**This study**
*C.kahawae*	Holotype	*Coffeaearabicae*	Kenya	12.5–19.0 × 4.0	8.0–9.5 × 5.5–6.5	Not observed	Waller et al. (1993)
*C.mengyinense*	Holotype	*Rosachinensis*	China	14.3 ± 1.1 × 5.3 ± 0.4	Not observed	Not observed	Mu et al. (2021)
*C.nanhuaensis*	Holotype	*Ageratinaadenophora*	China	14.0 ± 1.1 × 5.4 ± 0.4	8.0–14.0 × 5.0–8.0	25.0	Yu et al. (2022)
***C.peakense***	**Holotype**	***Juglansregia* L.**	**China**	**16.4 ± 1.4 × 4.9 ± 0.5**	**5.6–8.4 × 3.9–6.1**	57.2–152.9	**This study**
*C.siamense*	Holotype	*Coffeaarabica*	Thailand	10.2 ± 1.7 × 3.5 ± 0.4	4.7–8.3 × 3.5–5.0	Not observed	Prihastuti et al. (2009)
*C.viniferum*	Holotype	*Vitisvinifera*	China	13.8 ± 1.0 × 5.4 ± 0.4	6.5–10.5 × 4.8–6.3	Not observed	Peng et al. (2013)

#### ﻿ Colletotrichum peakense

Taxon classificationFungiGlomerellalesGlomerellaceae

Y. Zhang ter. & L. Zhang sp. nov.

207F42DE-3B29-5D29-BF9C-1A6B14E81B1C

MycoBank No: 848730

Fig. 4

##### Etymology.

Named after Beijing where the fungus was collected.

##### Description.

***Sexual morph*** not observed. ***Asexual morph*** developed on MEA. ***Conidiomata*** acervular, yellow, bearing conidial masses. ***Conidiophores*** hyaline, smooth-walled, septate and branched. ***Setae*** medium to dark brown, smooth to finely verruculose close to the tip, the tip rounded, 1–3 aseptate, 57.2–152.9 μm long. ***Conidiogenous cells*** 20–35.6 × 2.8–3.9 μm (mean SD = 26.1 ± 0.9 × 3.0 ± 0.1 μm, n = 20), subcylindrical, straight to curved. ***Conidia*** 13.5–20.5 × 3.1–5.9 μm (mean SD = 16.4 ± 1.4 × 4.9 ± 0.5 μm, L/W radio = 3.3, n = 100), hyaline, smooth-walled, subcylindrical, both ends round, 1–3-guttulate, contents granular. ***Appressoria*** 5.6–8.4 × 3.9–6.1 μm (mean SD = 6.7 ± 0.2 × 5.1 ± 0.1 μm, L/W radio = 1.3, n = 20), medium to dark brown, variable in shape, often smooth-walled, subglobose, ovate to broadly elliptical in outline.

**Figure 4.**
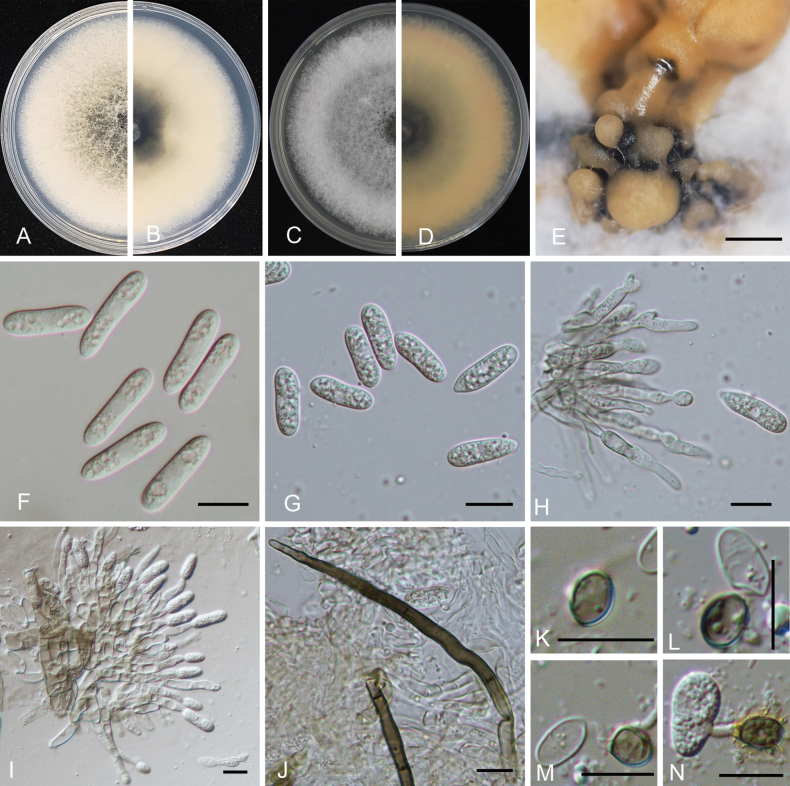
*Colletotrichumpeakense* (from ex-type CGMCC3.24308) **A, B** colonies and reverse after 7 days on PDA medium **C, D** colonies and reverse after 7 days on MEA medium **E** conidiomata **F, G** conidia **H, I** conidiophores **J** setae **K–N** appressoria. Scale bars: 500 μm (**E**); 10 μm (**F–N**).

***Asexual morph*** developed on PDA. ***Conidia*** 14.7–22.2 × 4.1–6.3 μm (mean SD = 17.4 ± 1.6 × 5.2 ± 1.6 μm, L/W radio = 3.3, n = 50), hyaline, smooth-walled, subcylindrical, both ends round, 1–3-guttulate, contents granular.

##### Culture characteristics.

Colonies on MEA, flat, with entire margin, hyaline, 68–78 mm diam. in 7 d. The colonies are round, aerial mycelium white or grey, floccose cottony; surface and reverse grey in the centre and white margin. Myxospores are orange. The colony diameter reached 76–80 mm on PDA. The colonies are round, aerial mycelium white or grey, floccose cottony; surface and reverse grey in the centre and white margin.

##### Additional specimens examined.

China, Beijing, Changping District, Heishanzhai Village, from leaf of *Juglansregia* L., 26 Aug 2021, Y. Zhang and L. Zhang (holotype HSY826-18; ex-type living culture, CGMCC3.24308. China, Beijing, Changping District, Heishanzhai Village, from leaf of *Juglansregia* L., 26 Aug 2021, Y. Zhang and L. Zhang (Paratype HSY826-18): living culture, CGMCC3.24307.

##### Notes.

Phylogenetic analysis of a concatenated five loci dataset indicated that the clade of *Colletotrichumpeakense* nested in the clade of *C.gloeosporioides* species complex and was closely related, but independent to *C.citrulli*, *C.dimorphum*, *C.gloeosporioides* and *C.nanhuaensis* (Cannon et al. 2008; Guo et al. 2022; Yu et al. 2022). Morphologically, *Colletotrichumpeakense* was distinguishable from *C.citrulli* by having longer setae and smaller appressoria (Guo et al. 2022) (Table 2), while from *C.dimorphum* by having longer conidia and longer setae (Yu et al. 2022) (Table 2), from *C.gloeosporioides* by having longer conidia (Cannon et al. 2008) (Table 2) and from *C.nanhuaensis* by having longer conidia and shorter appressoria (Guo et al. 2022) (Table 2). The PHI test (Φw = 1.0) detected no significant recombination between related isolates or species-related species (Fig. 2B).

### ﻿Pathogenicity tests on walnut tissues

**Figure 5.**
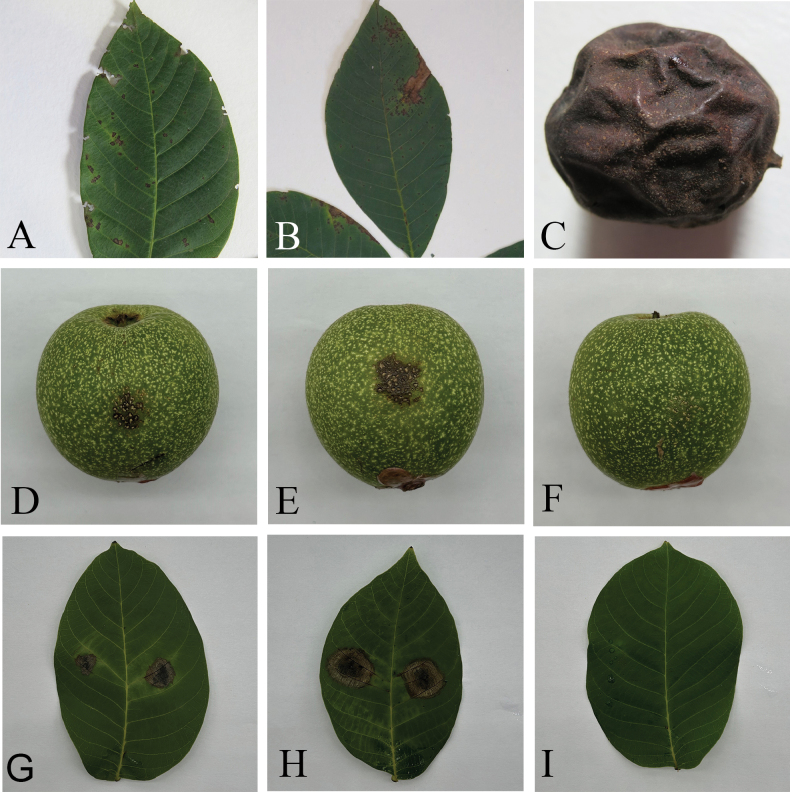
Anthracnose symptoms on walnut fruits and leaves caused by *C.peakense* and *C.juglandicola***A** anthracnose caused by *C.juglandicola* on leaf **B** anthracnose caused by *C.peakense* on leaf **C** anthracnose fruits caused by *C.juglandicola***D, G** symptoms of *C.juglandicola* (CGMCC3.24312) using spore suspension and non-wound inoculation methods after 10 days inoculation on walnut fruit (**D**) and leaf (**G**) **E, H** symptoms of *C.peakense* (CGMCC3.24308) using spore suspension and non-wound inoculation methods after 10 days inoculation on walnut fruit (**E**) and leaf (**H**) **F, I** symptoms resulting from sterilised water and non-wound inoculation methods after 10 days inoculation on walnut fruit (**F**) and leaf (**I**).

Pathogenicity tests were conducted to confirm Koch’s postulates on fruits and leaves of walnut for *C.juglandicola* and *C.peakense*. The symptom of circular, necrotic, sunken lesions on fruits and as circular, necrotic lesions on leaves after 10 days of inoculation with typical orange conidial masses were observed from the inoculated site, whereas all control fruits and leaves remained healthy (Fig. 5). For spore suspension and non-wound methods, both on fruits and leaves, the lesion diameter of *C.peakense* was significantly higher than *C.juglandicola* (*P* < 0.05) (Table 3). Furthermore, *Colletotrichum* isolates could consistently be re-isolated from symptomatic lesions, but never from control. Koch’s postulates were performed by successful pathogen re-isolation from all the necrotic fruits and leaves. The morphology and DNA sequences of these new isolates were consistent with the initial inoculation.

**Table 3.** Pathogenicity of *Colletotrichumjuglandicola* (CGMCC3.24312) and *Colletotrichumpeakense* (CGMCC3.24308) on walnut fruits and leaves using spore suspension as inoculum 10 days after inoculation.

Species	Walnut fruits inoculated with Spore suspension and non-wound ± SD (mm)	Walnut leaves inoculated with Spore suspension and non-wound ± SD (mm)
*Colletotrichumjuglandicola*	5.80 ± 1.27 b	8.90 ± 2.28 b
*C.peakense*	9.50 ± 1.0 a	16.79 ± 2.58 a
Non-inoculated control	0 ± 0 c	0 ± 0 c

Note: Data followed by different letters in each column are significantly different, based on HSD tests at the *P* < 0.05 level.

## ﻿Discussion

Phylogenetic analyses, based on five concatenated loci (*ACT*, *CHS-1*, *GAPDH*, ITS and *TUB2*), indicated that either *Colletotrichumjuglandicola* or *C.peakense* formed a distinct clade within the *C.gloeosporioides* complex, while sibling to other species (Fig. 1). On the phylogenetic tree, *C.juglandicola* is closely related to *C.citrulli*, *C.dimorphum*, *C.gloeosporioides* and *C.nanhuaensis* (Fig. 3). Morphologically, *C.juglandicola* can be readily distinguished from *C.citrulli*, *C.dimorphum*, *C.gloeosporioides* and *C.nanhuaensis*, based on its longer conidial size, presence or absence of setae and appressoria size (Table 2). Phylogenetically, *C.peakense* is closely related to *C.citrulli*, *C.dimorphum*, *C.gloeosporioides* and *C.nanhuaensis* (Fig. 4). *Colletotrichumpeakense* can be distinguishable from *C.citrulli*, *C.dimorphum*, *C.gloeosporioides* and *C.nanhuaensis* by its longer setae and smaller appressorial size (Table 2).

Thus far, 14 species of *Colletotrichum* have been reported from *Juglansregia* L., namely *C.acutatum*, *C.fioriniae*, *C.godetiae*, *C.juglandis* and *C.nymphaeae* of the Acutatum species complex, *C.aenigma*, *C.fructicola*, *C.gloeosporioides*, *C.kahawae*, *C.mengyinense*, *C.siamense* and *C.viniferum* of Gloeosporioides species complex, *C.liaoningense* of Magnum species complex and *C.sojae* of Orchidearum species complex (Simmonds 1966; Alvarez 1976; Gorter 1977; Pennycook 1989; Liu et al. 1995; Crous et al. 2000; Chen 2003; Cho and Shin 2004; Gadgil et al. 2005; Juhásová et al. 2005; Sreenivasaprasad and Talhinhas 2005; Kobayashi 2007; Qu et al. 2011; Damm et al. 2012; Zhu et al. 2014; Zhu et al. 2015; Wang et al. 2017, 2018; Da Lio et al. 2018; He et al. 2019; Savian et al. 2019; Wang et al. 2020; Wang et al. 2021, 2023; Luongo et al. 2022; Ma et al. 2022; Wei et al. 2022; Li et al. 2023), of which, *Colletotrichumacutatum*, *C.fioriniae*, *C.godetiae*, *C.juglandis* and *C.nymphaeae* differed from *C.juglandicola* and *C.peakense* by their acute-ended conidia (Damm et al. 2012). *Colletotrichumaenigma*, *C.fructicola*, *C.gloeosporioides*, *C.kahawae*, *C.mengyinense*, *C.siamense* and *C.viniferum* differed from *C.juglandicola* and *C.peakense*, by the size of conidia (Table 2). The conidia shape of *Colletotrichumjuglandicola* and *C.peakense* was comparable to *C.liaoningense*, while the longer conidia and longer appressoria size were distinguishable from the latter (Diao et al. 2017) (Table 2). *Colletotrichumjuglandicola* and *C.peakense* were distinguishable from *C.sojae* by their shorter appressoria (Damm et al. 2019) (Table 2).

Pathogenicity tests indicated that both *Colletotrichumjuglandicola* and *C.peakense* cause anthracnose disease in walnut fruits and leaves. Both on fruits and leaves, the virulence of *C.peakense* was more severe than *C.juglandicola* (*P* < 0.05). *Colletotrichumgloeosporioides* had been reported more severe than most other species in Beijing, which was supported by the current study in that *C.gloeosporioides* was more severe than *C.juglandicola* (12.33 ± 0.29 mm in 4 days vs. 8.90 ± 2.28 mm in 10 days) (Li et al. 2023).

Both *Colletotrichumjuglandicola* and *C.peakense* belong to the *C.gloeosporioides* species complex, which has been reported as one of the most important pathogens worldwide and has infected at least 1,000 plant species (Phoulivong et al. 2010). *Colletotrichumgloeosporioides* species complexes could be either broad or narrow host ranges (Crouch et al. 2014; Liu et al. 2022). It appears that some species of *Colletotrichum*, such as *C.horii* (on persimmon) and *C.kahawae* (on coffee) may be restricted to certain hosts genera or families, while some others may have a wide range of hosts. For instance, *C.fructicola* has been reported from walnut, coffee, chilli, longan, shine muscat, papaya and tea (Prihastuti et al. 2009; Yang et al. 2009; Sharma and Shenoy 2014; Wang et al. 2018; Lim et al. 2020; Lin et al. 2021). To summarise, in Beijing, *C.juglandicola* and *C.peakense*, two species new to science, were the causal agents of walnut anthracnose.

## Supplementary Material

XML Treatment for Colletotrichum juglandicola

XML Treatment for Colletotrichum peakense

## ﻿Additional information

### Author ORCIDs

Lin Zhang https://orcid.org/0009-0002-6325-1440

Yue-Qi Yin https://orcid.org/0009-0009-0756-5075

Li-Li Zhao https://orcid.org/0000-0003-1451-3301

Yu-Qing Xie https://orcid.org/0009-0009-8720-3276

Ying Zhang https://orcid.org/0000-0001-8817-6032

### Data availability

All of the data that support the findings of this study are available in the main text.
